# Exploring “Equigenesis” in the Associations Between Green Space and Kidney Health Among Middle-Aged and Older Adults Using Street View Data

**DOI:** 10.1093/geroni/igad130

**Published:** 2023-11-23

**Authors:** Ruoyu Wang, Guoping Dong, Mengqiu Cao, Yang Zhou, Guang-Hui Dong

**Affiliations:** Institute of Public Health and Wellbeing, University of Essex, Essex, UK; School of Accounting, Guangzhou Huashang College, Guangzhou, China; School of Architecture and Cities, University of Westminster, London, UK; State Environmental Protection Key Laboratory of Environmental Pollution Health Risk Assessment, South China Institute of Environmental Sciences, Ministry of Environmental Protection, Guangzhou, China; Guangdong Provincial Engineering Technology Research Center of Environmental and Health Risk Assessment, Department of Occupational and Environmental Health, School of Public Health, Sun Yat-Sen University, Guangzhou, China

**Keywords:** Healthy aging, Inequalities, Kidney failure, Machine learning, Visible greenery

## Abstract

**Background and Objectives:**

This study systematically explores the association between community green space and preventing kidney failure among middle-aged and older adults in China, using street view data.

**Research Design and Methods:**

The 33 Chinese Community Health Study was used to conduct the analysis. We used street view data to assess street view green space (SVG) exposure and clearly distinguished the difference between grass (SVG-grass) and trees (SVG-tree). The normalized difference vegetation index (NDVI) was also used. Kidney failure was defined as a serum creatinine concentration of above 177 mol/L. We used multilevel logistic regression models (controlled for a series of covariates) to examine the associations between SVG and the odds of middle-aged and older adults having kidney failure. We also tested whether middle-aged and older adults from socioeconomically disadvantaged groups are likely to derive greater benefits from the effects of green space (“equigenesis”).

**Results:**

The results showed that both SVG (OR = 0.353; 95% CI = 0.171–0.731) and SVG-trees (OR = 0.327; 95% CI = 0.146–0.736) were negatively associated with the likelihood of middle-aged and older adults experiencing kidney failure, but there was no significant evidence of any links between either SVG-grass (OR = 0.567; 95% CI = 0.300–1.076) or the NDVI (OR = 0.398; 95% CI = 0.237–1.058) and kidney failure. Furthermore, the moderation analysis indicated that income and educational attainment have a moderating effect on the association between green space and the improvement of kidney health, which suggests that green space has greater positive effects on the kidney health of disadvantaged groups.

**Discussion and Implications:**

To reduce inequalities in relation to kidney disease through urban planning, policymakers are advised to provide more visual green space—especially trees—within the community and to focus in particular on socioeconomically disadvantaged population groups.

**Translational Significance:** There is a lack of empirical studies on the association between green space and kidney health in middle-aged and older adults in developing countries. The study found that there was an association between street-level grass (but not trees) and a reduced likelihood of kidney failure in middle-aged and older adults. This association was more pronounced for socioeconomically disadvantaged groups, which indicates that street-level green space may help to reduce the effects of socioeconomic inequalities in kidney disease among middle-aged and older adults.

Chronic kidney disease (CKD) has become a significant public health issue in recent years (Carney, 2020). In 2017—the latest date for which figures are available—the global prevalence of CKD was reported to be 9.1% (697.5 million cases; Bikbov et al., 2020; Carney, 2020). From 1990 to 2017, the global prevalence of CKD among all age groups increased by 29.3% (Bikbov et al., 2020; Carney, 2020). In 2017, CKD was the 12th leading cause of death, accounting for 1.2 million deaths, 35.8 million disability-adjusted life years, and 4.6% of all-cause mortality (Bikbov et al., 2020; Carney, 2020). In China, the overall prevalence of CKD was 10.8% in 2012 (Zhang et al., 2012), meaning that the country accounted for nearly one-sixth (132.3 million) of the total number of cases worldwide in 2017 (Bikbov et al., 2020; Carney, 2020). CKD is more prevalent among older adults than younger adults, which makes the older population more vulnerable (Bikbov et al., 2020; Carney, 2020). Worse still, the population in China has been increasingly aging in recent years, at a more rapid rate than most other countries around the world (World Health Organization, 2015). It is estimated that between 2010 and 2040, China’s older population (people aged over 60) will grow from 177.6 million to 402 million (The State Council of the People’s Republic of China, 2017; Wu & Dang, 2013).

Existing evidence suggests that the built environment plays a significant role in the prevalence of CKD (McClellan et al., 2012). As an important health-related component of the built environment, green space has attracted considerable attention in the literature, as it can be easily addressed through urban planning policy (Chaudhury & Xu, 2022; Liang et al., 2022; Liu et al., 2023; Lu et al., 2022; Park et al., 2021; Shen, 2022). For example, Liang et al. (2022) found that residential greenness was associated with a decreased prevalence of CKD in China. Liu et al. (2023) also suggested that there was an inverse association between residential green space and CKD in the UK. Meanwhile, Park et al. (2021) found that residential greenness reduced the risk of all-cause mortality from CKD and the progression of CKD to end-stage renal disease in Korea. Shen (2022) also pointed out that the proportion of space covered by greenery is negatively associated with CKD prevalence. There are several pathways linking green space to CKD (Liang et al., 2022; Liu et al., 2023; Park et al., 2021; Shen, 2022). First, there is an increasing body of literature evidencing the beneficial effects of neighborhood green space on residents’ health because it reduces their exposure to deleterious environmental factors such as air pollution, heatwaves, and noise (Grzędzicka, 2019; Zawadzka et al., 2021). Second, encouraging physical activity and facilitating neighborhood social contact also offers a way of increasing the capacity for exposure to green space, which has beneficial effects on human health (Wang et al., 2020, 2021). Third, some scholars have argued that green space plays a key role in strengthening the immune system, which is important in the prevention of chronic diseases (Kuo, 2015; Q. Li et al., 2008).

As well as having a direct protective effect on health, green space has also been found to improve health equality (equigenesis), which means that socioeconomically disadvantaged groups may be able to benefit more from green space, thus reducing the gap between them and their wealthier counterparts in terms of health inequalities (Mitchell et al., 2015; Pearce et al., 2015). A possible explanation for this finding is that socioeconomically disadvantaged groups usually have to rely more on public facilities such as public green space, due to a lack of health-related resources, and being unable to afford private medical care (Wang et al., 2022). However, existing empirical evidence for the heterogeneous effect of green space on CKD is inconsistent (Liang et al., 2022; Liu et al., 2023). Some studies have found that the effect of green space on CKD is stronger for socioeconomically disadvantaged groups (Liu et al., 2023), whereas others have claimed that the effect of green space on CKD is more pronounced among socioeconomically advantaged groups (Liang et al., 2022). For example, Liang et al. (2022) suggested that the protective effects of green space on CKD prevalence are stronger among people of a higher socioeconomic status, whereas Liu et al. (2023) found the effect of green space on helping to prevent CKD to be greater for residents living in more deprived neighborhoods. Some scholars have argued that these inconsistencies regarding the “equigenesis” of green space may be partly due to how green space is measured (Feng & Astell-Burt, 2017; Wang et al., 2022). Most of the existing evidence has been obtained using remote sensing or land use data to measure green space exposure and thus may have ignored the influence of street-level visible green space (Wang et al., 2020, 2021). The lack of attention that has been paid to street-level visible green space by most previous studies is mainly due to methodological limitations (Wang et al., 2020, 2021). They have tended to use either survey questionnaires or field audits to assess green space, both of which are time-consuming and expensive (Wang et al., 2020, 2021). In recent years, with the development of machine learning, scholars have increasingly begun to use street-view images to measure green space that is visible at street level (Wang et al., 2020, 2021). Therefore, it is feasible to use a method that combines street view images with machine learning to study the association between green space and CKD.

As discussed earlier, there are several gaps in the literature. Although it is clear that an association exists between green space and CKD in general, there is still no empirical evidence to show whether green space can also play a role in reducing the likelihood of kidney failure. Although CKD (e.g., kidney failure) is more prevalent among middle-aged and older adults than younger adults, scant attention has been paid to the associations between green space and kidney disease in middle-aged and older adults. As noted earlier, existing literature has mainly relied on using land use or remote sensing data to measure green space exposure, which may have resulted in exposure to green space that is visible at street level being overlooked. Finally, evidence has shown that green space can help to reduce health inequalities (“equigenesis”). However, it is still unclear whether green space can also reduce inequalities specifically relating to kidney failure among members of the population who are older than 60. Therefore, in this study, we first aim to examine the association between exposure to community green space that is visible at street level and kidney failure in middle-aged and older adults using street-view data. Second, we investigate whether socioeconomically disadvantaged groups of middle-aged and older adults may derive more benefits from exposure to green space (“equigenesis”).

## Data and Methods

### Survey Data

The 33 Chinese Community Health Study (CCHS), conducted in 2009 and 2010, was used to carry out the analysis (Yang et al., 2017). The 33 CCHS used a four-stage cluster sampling method to recruit participants from three major cities in northern China that were chosen out of a possible 14—Shenyang, Anshan, and Jinzhou. A total of 33 communities (*shequ*) were then randomly selected from 11 districts in the three sampled cities. Next, households from each community were also randomly selected, and finally, one adult respondent was chosen from each household. After the data had been cleaned and any missing information excluded, a total of 2,154 middle-aged and older adults (>50 years) were included in the final analysis. The study was conducted according to the principles stipulated by the Declaration of Helsinki and all procedures were approved by the Human Studies Committee of Sun Yat-Sen University (Identification code: SYSU016). We also obtained informed written consent from all the participants.

### Kidney Failure

After they had fasted overnight, urine samples were collected from all participants the following morning. The urine tests were carried out at the institutional laboratories of local community health service centers. Following existing studies set in China (Zhang et al., 2012), kidney failure was defined as having a serum creatinine level of >177mol/L. Although the cutoff point of serum creatinine levels used to define kidney failure varies between different studies (e.g., in one UK study, kidney failure was defined as having a clinical laboratory serum creatinine level of >180 mol/L), existing meta-studies suggest that the operational definition of kidney failure does not significantly affect the results (Clase, 2011; Tangri et al., 2011).

### Green Space Exposure

Tencent street-view images (https://map.qq.com/) collected in 2011 and 2012 were used to measure exposure to street-view green space (SVG). We used open street map (https://www.openstreetmap.org) to construct the sampling points for the street-view images, which were 100 m apart from each other. Following the existing literature (Wang et al., 2020, 2021), four images (0, 90, 180, and 270°) were collected for each sampling point to provide a comprehensive view of the street-level environment. In total, 666,758 street-view images were collected within the research area. We used the ADE20K data set (Zhou et al., 2019) to train the model and a fully convolutional neural network (FCN-8s; Long et al., 2015) to carry out the image segmentation. The validation process was conducted by comparing the model output from the FCN-8s and manually marking the segmentation images (ADE20K data set) using a 5-fold cross-validation process. The overall accuracy of the trained model was above 0.85 in terms of detecting vegetation (trees and grass). Previous literature has suggested that trees and grass have heterogeneous effects on health outcomes (Wang et al., 2020), so we calculated both exposure to trees at street-view level (SVG-tree) and exposure to grass at street-view level (SVG-grass). SVG-tree/SVG-grass exposure per sampling point represents the ratio of the number of tree/grass pixels per image summed over the four cardinal directions to the total number of pixels per image summed over the four cardinal directions. SVG was computed similarly but took both trees and grass into account. We calculated the mean values of all the sampling points within the 1,000-m circular buffers of respondents’ home addresses, as the buffers were designed to represent 10–15 min walking distance from their residences (Merriam et al., 2017).

We also used the normalized difference vegetation index (NDVI; Tucker, 1979) to measure respondents’ overall exposure to green space. We collected cloud-free Landsat 5 Thematic Mapper satellite images taken during August 2010 (the greenest month). The NDVI value was calculated using the following formula: (NIR − VIS)/ (NIR + VIS), where NIR is the reflectance in the near-infrared band and VIS is the reflectance in the visible region. We also calculated SVG as the mean value of all the pixels within the 1,000-m residential circular buffer.

### Covariates

Following previous studies (Liang et al., 2022; Liu et al., 2023; Park et al., 2021; Shen, 2022), we controlled for several covariates relevant to the association between green space and kidney health namely gender (men vs. women), age (years), educational attainment (primary school and below vs. high school and above), annual household income (<30,000 Yuan vs. ≥30,000 Yuan), employment category (white-collar worker vs. others), controlled low-calorie and low-fat diet (yes = controlled low-calorie and low-fat diet every day vs. no = others), physical activity behavior (active = takes regular exercise vs. inactive = others), and BMI (body mass index). We also included the annual average levels of particles ≤2.5 µm in aerodynamic diameter (PM_2.5_) and nitrogen oxides (NO) to control for the effects of air pollution. They were calculated using a land-use regression model based on ground-monitoring data, aerosol optical depth data from NASA Moderate Resolution Imaging Spectroradiometers, vegetation data, land use information, meteorological data, and other spatial predictors. In addition, we controlled for neighborhood-level population density (persons/km^2^) and neighborhood gross domestic product (GDP) per capita (Chinese Yuan), using data obtained from the Resource and Environment Science and Data Center in 2010. More information about these can be found in previous studies (Yang, Guo, et al., 2019). The summary statistics of the variables are shown in Table 1. We used Student’s *t*-tests or chi-square tests to examine the differences in individual-level characteristics between participants with kidney failure and those without kidney failure (Supplementary Table 1).

**Table 1.** Summary Statistics of Variables (*n* = 2,154)

Variables	*n* (%)	Mean (*SD*)	Median (Q1–Q3)
Gender			
Men	1,002 (46.52)		
Women	1,152 (53.48)		
Age (years)		59.40 (6.53)	
Educational attainment			
Primary school and below	792 (36.77)		
High school and above	1,362 (63.23)		
Annual household income			
<30,000 Yuan	1,797 (83.43)		
≥30,000 Yuan	357 (16.57)		
Employment category			
White-collar worker	441 (20.47)		
Others	1,713 (79.53)		
Controlled low-calorie and low-fat diet			
Yes	137 (6.36)		
No	2,017 (93.64)		
Physical activity behavior			
Active	802 (37.23)		
Inactive	1,352 (62.77)		
Body mass index (kg/m^2^)		25.36 (3.66)	
NO (μg/m^3^)		3.95 (0.60)	
PM_2.5_ (μg/m^3^)		83.41 (15.78)	
Kidney failure			
Yes (serum creatinine > 177mol/L)	38 (1.76)		
No	2,116 (98.24)		
SVG (0–1)			0.103 (0.087–0.122)
NDVI (0–1)			0.299 (0.249–0.383)
SVG-tree (0–1)			0.101 (0.086–0.117)
SVG-grass (0–1)			0.003 (0.002–0.005)
Neighborhood population density (persons/km^2^)		17,144.959 (15,023.505)	
Neighborhood GDP per capita (Chinese Yuan)		16,388.951 (31,582.739)	

*Notes*: GDP = gross domestic product; NDVI = normalized difference vegetation index; NO = nitrogen oxides; PM_2.5_ = particles ≤ 2.5 µm in aerodynamic diameter; SVG = Street-view green space.

### Statistical Analysis

To assess the associations between community green space exposure and kidney failure in middle-aged and older adults, we modeled several multilevel logistic regressions (Guo & Zhao, 2000). The test for the variance inflation factors (VIF = 1.38) showed that there was no potential multicollinearity identified between the predictors. The intraclass correlation coefficient (ICC) for the null model predicting the odds of having kidney failure was 0.12, which means that living within the same community accounted for 12% of the total variation in respondents’ odds of having kidney failure. Consequently, it was necessary to use multilevel models.

The first step involved regressing the odds of having kidney failure using different green space metrics and covariates (Models 1–4). We then estimated the moderating effect of educational attainment (Models 5–8) and income (Models 9–12), respectively. With regard to the moderation analysis, we mainly focused on the direction and significance level of the interaction terms. We designated the low socioeconomic status (SES) group as the reference group (i.e., respondents with lower levels of educational attainment and income), so if the interaction terms were opposite in direction to the green space metrics, that would mean that green space has less influence on people of higher SES and a greater effect on people of lower SES. In this case, the “equigenesis” theory is supported by the findings. We also stratified the analysis by educational attainment and household income to further show the heterogeneous effect of green space (Supplementary Figures 2 and 3). We defined statistical significance as *p* < .05 and the results are presented in the form of odds ratios (OR) with 95% confidence intervals (CI). The models were adjusted for gender, age, educational attainment, annual household income, employment category, diet, physical activity behavior, BMI, and PM_2.5_, NO neighborhood-level population density, and GDP per capita, as described earlier. The analyses were conducted using Stata version 15.1 (StataCorp LP, College Station, TX).

## Results

Table 2 shows the results regarding the association between green space exposure and kidney failure. Model 1 indicated that SVG was negatively associated with the odds of having kidney failure (OR = 0.353, 95% CI: 0.171–0.731). Model 2 showed that SVG-tree was negatively associated with the odds of having kidney failure (OR = 0.327, 95% CI: 0.146–0.736). Models 3 and 4 revealed that there is no evidence that SVG-grass (OR = 0.567, 95% CI: 0.300–1.076) or NDVI (OR = 0.398, 95% CI: 0.237–1.058) was associated with the odds of having kidney failure. Overall, males were more likely to have kidney failure than women. As physical activity, BMI, and air pollution are likely to be mediators, and could therefore cause potential bias, we then excluded these two variables and reran the model as a sensitivity analysis (Supplementary Figure 1A). In addition, we excluded two neighborhood-level variables (density and GDP) and two individual-level variables (employment category and diet) to test the robustness of our results (Supplementary Figure 1B). Despite some differences in magnitude, the associations between SVG and kidney failure remained the same for the less adjusted model (Supplementary Figure 1).

**Table 2.** Results of the Multilevel Models Used to Examine the Association Between Green Space Exposure and Kidney Failure (*n* = 2,154)

Variable	Model 1	Model 2	Model 3	Model 4
	OR (95% CI)	OR (95% CI)	OR (95% CI)	OR (95% CI)
Male (ref = women)	2.965**(1.265–6.948)	2.967**(1.268–6.942)	3.077***(1.342–7.057)	3.008***(1.354–6.681)
Age	0.999(0.940–1.063)	0.999(0.940–1.063)	1.003(0.943–1.065)	1.003(0.944–1.066)
High school or above (ref = primary school or below)	1.594(0.617–4.113)	1.618(0.627–4.170)	1.501(0.584–3.850)	1.629(0.640–4.144)
Household income ≥ 30,000 Yuan (ref = household income < 30,000 Yuan)	1.605(0.708–3.638)	1.613(0.711–3.659)	1.721(0.761–3.888)	1.659(0.736–3.736)
White-collar worker (ref = Others)	13.676***(6.358–29.415)	13.601***(6.321–29.261)	14.440***(6.724–31.006)	14.255***(6.693–30.361)
Controlled low-calorie and low-fat diet				
Yes (ref = No)	1.317(0.433–4.005)	1.319(0.433–4.015)	1.239(0.413–3.717)	1.322(0.447–3.905)
Physically active (ref = physically inactive)	0.512(0.229–1.144)	0.516(0.231–1.153)	0.508(0.228–1.132)	0.533(0.240–1.185)
Body mass index	0.929(0.828–1.043)	0.929(0.828–1.043)	0.935(0.835–1.047)	0.924(0.824–1.037)
NO	1.363(0.601–3.091)	1.301(0.574–2.948)	1.344(0.576–3.139)	1.356(0.643–2.856)
PM_2.5_	1.001(0.967–1.036)	1.002(0.968–1.037)	1.001(0.968–1.035)	0.998(0.967–1.031)
Neighborhood population density	0.999(0.999–1.000)	0.999(0.999–1.000)	0.999(0.999–1.000)	0.999(0.999–1.000)
Neighborhood GDP per capita	0.999**(0.999–0.999)	0.999**(0.999–0.999)	0.999**(0.999–0.999)	0.999**(0.999–0.999)
SVG	0.353***(0.171–0.731)			
SVG-tree		0.327***(0.146–0.736)		
SVG-grass			0.567*(0.300–1.076)	
NDVI				0.398*(0.237–1.058)
Log likelihood	−147.535	−148.021	−151.050	−148.420
AIC	325.069	326.042	332.100	324.841

*Notes*: AIC = Akaike information criterion; CI = confidence interval; GDP = gross domestic product; NDVI = normalized difference vegetation index; NO = nitrogen oxides; OR = odds ratio; PM_2.5_ = particles ≤ 2.5 µm in aerodynamic diameter; SVG = street-view green space; .

*p < .10, **p < .05, ***p < .01.

Table 3 shows the moderating effect of educational attainment on the association between green space exposure and kidney failure. Model 5 indicates that educational attainment has a positive moderating effect on the association between SVG and the odds of having kidney failure (OR = 1.284, 95% CI: 1.094–3.021). This means that the effect of SVG on the likelihood of having kidney failure is lower for respondents with an educational attainment level of high school or above than for those with an educational attainment level of primary school or below. Hence, Model 6 indicates that educational attainment also has a positive moderating effect on the association between SVG-tree and the odds of having kidney failure (OR = 1.196, 95% CI: 1.023–3.038). This means that the effect of SVG-tree on the likelihood of having kidney failure is lower for respondents with an educational attainment level of high school or above than for their counterparts with an educational attainment level of primary school or below. However, there is no evidence that educational attainment moderates the association between SVG-grass or NDVI and kidney failure.

**Table 3.** Results of the Multilevel Models Used to Examine the Association Between Green Space Exposure and Kidney Failure (Disparities in Educational Attainment; *n* = 2,154)

Variable	Model 5	Model 6	Model 7	Model 8
	OR (95% CI)	OR (95% CI)	OR (95% CI)	OR (95% CI)
SVG	0.367** (0.073–0.847)			
SVG-tree		0.282** (0.052–0.825)		
SVG-grass			0.658 (0.204–2.113)	
NDVI				0.720 (0.213–2.429)
SVG × High school or above (ref = primary school or below)	1.284** (1.094–3.021)			
SVG-tree × High school or above (ref = primary school or below)		1.196** (1.023–3.038)		
SVG-grass × High school or above (ref = primary school or below)			0.820 (0.214–3.146)	
NDVI × High school or above (ref = primary school or below)				0.466 (0.114–1.902)
Log likelihood	−148.008	−148.001	−151.010	−147.887
AIC	328.016	328.002	334.020	325.773

*Notes*: AIC = Akaike information criterion; CI = confidence interval; NDVI = normalized difference vegetation index; OR = odds ratio; SVG = street-view green space. Models were fully adjusted as was the case in Table 2.

**p < .05.

Table 4 shows the moderating effect of household income on the association between green space exposure and kidney failure. Model 9 indicates that household income has a positive moderating effect on the association between SVG and the odds of having kidney failure (OR = 1.072, 95% CI: 1.003–3.833). This means the effect of SVG on the odds of having kidney failure is smaller for respondents with a household income of ≥30,000 Yuan than for those with a household income of <30,000 Yuan. Model 10 shows that household income also has a positive moderating effect on the association between SVG-tree and the likelihood of having kidney failure (OR = 1.086, 95% CI: 1.007–4.413). This means that the effect of SVG-tree on the odds of having kidney failure is smaller for respondents with a household income of ≥30,000 Yuan than for those with a household income of <30,000 Yuan. However, there is no evidence that household income moderates the association between SVG-grass or NDVI and kidney failure.

**Table 4.** Results of the Multilevel Models Used to Examine the Association Between Green Space Exposure and Kidney Failure (Disparities in Income; *n* = 2,154)

Variable	Model 9	Model 10	Model 11	Model 12
	OR (95% CI)	OR (95% CI)	OR (95% CI)	OR (95% CI)
SVG	0.361** (0.163–0.801)			
SVG-tree		0.336** (0.139–0.816)		
SVG-grass			0.549 (0.265–1.136)	
NDVI				0.319 (0.148–1.689)
SVG × Household income ≥ 30,000 Yuan (ref = household income < 30,000 Yuan)	1.072** (1.003–3.833)			
SVG-tree × Household income ≥ 30,000 Yuan (ref = household income < 30,000 Yuan)		1.086** (1.007–4.413)		
SVG-grass × Household income ≥ 30,000 Yuan (ref = household income < 30,000 Yuan)			1.150 (0.286–4.622)	
NDVI × Household income ≥ 30,000 Yuan (ref = household income < 30,000 Yuan)				2.402 (0.622–9.272)
Log likelihood	−147.526	−148.010	−151.031	−147.663
AIC	327.051	328.020	334.061	325.326

*Notes*: AIC = Akaike information criterion; CI = confidence interval; NDVI = normalized difference vegetation index; OR = odds ratio; SVG = street-view green space. Models were fully adjusted as was the case in Table 2.

**p < .05.

## Discussion

This study extends previous research in several respects. First, it enhances our knowledge about the kidney health of middle-aged and older adults which contributes to research on the topic of healthy aging. Second, this study makes a significant methodological contribution. It is the first study to use street-view data to assess exposure to visible green space and link it to the likelihood of kidney failure. Third, the study also contributes to the “equigenesis” theory in the Chinese context, by demonstrating that green space has the potential to reduce health inequalities in relation to kidney disease.

The major finding of this study is that SVG-tree was negatively associated with the odds of experiencing kidney failure among middle-aged and older adults. First, existing evidence has suggested that street trees are important in blocking sound waves and air pollutants from road traffic, which is important in the prevention of chronic diseases (Aerts et al., 2020; Bloemsma et al., 2019; Diener & Mudu, 2021; Dzhambov, Hartig, et al., 2018; Dzhambov, Markevych et al., 2018; Wang et al., 2020; Yuchi et al., 2020). For example, the leaves on street trees can prevent air pollutants from road traffic forming dense concentrations within a neighborhood, and therefore act as a buffer between air pollutants and humans inhaling them (Selmi et al., 2016). Street trees can also reduce traffic noise through either the diffraction, destruction, or absorption of sound waves (acoustic effect; Jang et al., 2015) or by mitigating the psychological stress caused by noise (psychological effect; Nang Li et al., 2012). Second, previous studies have found that street trees encourage more physical activity (Wang et al., 2021), which is important for kidney health. Street trees can provide shade for walkers or cyclists from the sunlight (Li & Ratti, 2018; Li et al., 2018), so residents are more likely to engage in more physical activity and for a longer duration in neighborhoods with more trees (Hunter et al., 2019; Lu, 2019). Hence, engaging in physical activity in green spaces, for example in areas where there are street trees, can increase the resultant health benefits (Mitchell, 2013), so it is likely that walking or cycling in an outdoor environment containing street trees will have a more positive effect on kidney health than indoor activities. Third, existing literature has pointed out that trees also provide a pleasant open space for socializing, and can thus facilitate more social contact (Hong et al., 2018; Samsudin et al., 2022). Therefore, middle-aged and older adults with higher residential proximity to green space are more likely to be part of a socially cohesive neighborhood (Hong et al., 2018; Samsudin et al., 2022) and may also get more social support, which is beneficial for their kidney health. Last, the density of street trees has also been found to have a restorative effect on local residents (Jiang et al., 2014; Jiang et al., 2016), so it is possible that middle-aged and older adults living in neighborhoods with more street trees feel calmer and less stressed and are thus more likely to have better kidney health. The microbial diversity hypothesis suggests that trees help to boost humans’ natural defenses against disease, by increasing the turnover of beneficial bacterial species (e.g., natural killer cells) in the gut, and levels of intracellular anticancer proteins, as well as significantly decreasing the concentration of adrenaline in the urine, which is beneficial for kidney function (Kuo, 2015; Li et al., 2008). However, we did not find any evidence that SVG-grass was associated with the likelihood of middle-aged and older adults experiencing kidney failure. One possible explanation is that grassland usually covers a relatively large area, so it is hard to maintain and often tends to be of low quality in Chinese neighborhoods (Xiao et al., 2016, 2017). Existing studies have pointed out that the quality of green space is vital for it to be beneficial to health, and that poorly maintained green space can actually make people feel less safe, which could even have a negative effect on their health (Bogar & Beyer, 2016; Maas et al., 2009). Another possible explanation is that grass is low in height compared with trees, so it is less visible to pedestrians walking along the street. Hence, the visibility of green space also plays an important role in residents’ health, because it directly affects the restorative effect that green space has (Wang et al., 2020, 2021). Furthermore, in the Chinese context, grassland within a residential neighborhood is often used by a management company for storing equipment or may not even be open to local residents, in which case it does not function as a publicly available open space (Xiao et al., 2016, 2017). There is no evidence to suggest that NDVI was related to the odds of middle-aged and older adults experiencing kidney failure. NDVI can provide an indication of the presence of large green spaces such as parks (Lu, 2019), but these green areas may not be publicly available within the neighborhoods where our research was conducted, which may partly explain the insignificant association between NDVI and kidney failure. In addition, the spatial resolution of the NDVI metric used may not have been precise enough to accurately reflect the quantity of green space, so using NDVI to measure the association between green space and kidney health may result in some inaccuracies.

The moderation analysis indicated that both educational attainment and income moderate the association between SVG and kidney failure, which means middle-aged and older adults with lower incomes or levels of educational attainment were more influenced by SVG. First, middle-aged and older adults earning higher incomes can afford a better standard of medical care, such as regular kidney checks and supplements (Dubay & Lebrun, 2012). This underlines the importance of SVG exposure for middle-aged and older adults on lower incomes, given that it is a free and publicly available health-promoting resource. Hence, middle-aged and older adults on higher incomes might prefer to make use of better quality, private green space further away from residential neighborhoods, but economically disadvantaged groups have no choice but to try to obtain health benefits from SVG exposure within their neighborhood. Second, middle-aged and older adults with lower levels of educational attainment appear to derive greater benefits from SVG, and this may be explained by their lack of health-related knowledge and social capital. For instance, middle-aged and older adults with higher levels of educational attainment usually have more knowledge about health matters, which can help them live a healthier lifestyle and make choices that are likely to have more positive effects on their health, such as engaging in more frequent physical activity, but those with lower levels of educational attainment may not be as knowledgeable about these matters and thus are more likely to engage in unhealthy behaviors (e.g., to be physically inactive), with deleterious effects for kidney health (Walsemann et al., 2013). Therefore, as a free source of health benefits, green space that is visible at the street level can facilitate healthier behavior such as physical activity, and thus is particularly important for middle-aged and older adults with lower levels of educational attainment. Middle-aged and older adults with higher levels of educational attainment usually have more social capital, which can also have a positive effect on their health, because their social networks are more likely to be comprised of their socioeconomically advantaged peers, which enables them to access more health-related information and resources (Choi et al., 2014; Hu et al., 2014; Riumallo-Herl et al., 2014). However, middle-aged and older adults with lower levels of educational attainment may not have sufficient social capital to access this kind of health-related information and resources, so green space can provide a means for them to socialize and build up their social networks, which may in turn be useful in terms of facilitating access to more health-related information (Thompson et al., 2013).

The following limitations of this study should be noted. First, the study was based on cross-sectional data, so we can only infer the correlation between green space and its role in reducing the likelihood of kidney failure, rather than any causality between them. Also, as it is a cross-sectional study and the older adults who participated may not have had complete freedom of choice regarding their residential locations, there is a possibility of residential self-selection bias, which could affect the analysis. Second, kidney failure was assessed by the clinical laboratory team, and measured based on the serum creatinine concentration for each individual. The threshold of serum creatinine >177 mol/L was set by the clinical laboratory team based on existing evidence and it was not possible for us to access the information about the actual serum creatinine level of each individual. Therefore, measurement bias and heterogeneity between participants could have some effect on the operational definition of kidney failure, which may cause a potential bias with regard to our statistical analysis. It is possible that the number of people categorized as having kidney failure might be too low which could have an influence on the statistical power of the analysis, and thus potentially weaken the scientific evidence regarding the association between SVG and kidney failure. Third, there are certain drawbacks associated with using street-view data. For example, the image segmentation algorithm that the street-view data uses was obtained by training it with a comprehensive data set, which might differ from our research area. Additionally, the data were collected during a specific period, so they may not be able to provide an accurate reflection of green space throughout the whole year. Fourth, because the health data were collected in 2009 and 2010, and the street-view data were collected in 2011 and 2012, this mismatch between the collection times could lead to a potential bias. Lastly, the study only assessed exposure to green space within participants’ residential neighborhoods. However, middle-aged and older adults may have some mobility restrictions and thus may exercise in places other than green spaces within their residential neighborhoods.

## Conclusion

This is the first study to explore the effect of green space on reducing the likelihood of kidney failure among middle-aged and older adults in China using street-view data. It also tested whether socioeconomically disadvantaged middle-aged and older adults may benefit more from exposure to green space (“equigenesis”). The results showed that there was a negative association between both SVG and SVG-tree and the odds of middle-aged and older adults having kidney failure, but there was no significant evidence of any link between SVG-grass or NDVI and kidney health. Furthermore, the moderation analysis indicated that income and educational attainment have a moderating effect on the association between green space and kidney failure; thus, socioeconomically disadvantaged groups derive greater benefits for their kidney health from exposure to green space. To achieve the goal of promoting better kidney health and reducing health inequalities regarding kidney disease through urban planning, policymakers and planners are advised to provide more green space that is visible at street level—especially trees—within the community and to focus in particular on socioeconomically disadvantaged groups.

## Supplementary Material

igad130_suppl_Supplementary_Material
