# Supplementary material for: Exploring “Equigenesis” in the Associations Between Green Space and Kidney Health Among Middle-Aged and Older Adults Using Street View Data
Source: Innov Aging. 2023 Nov 23;8(1):igad130. doi: 10.1093/geroni/igad130 (PMC10790735; doi:10.1093/geroni/igad130)
Supplement: igad130_suppl_Supplementary_Material [file igad130_suppl_supplementary_material.docx]

*Innovation in Aging* Online Supplementary Material: Ruoyu Wang, Guoping Dong, Mengqiu Cao, Yang Zhou, Guang-Hui Dong. Exploring ‘equigenesis’ in the associations between green space and kidney health among middle-aged and older adults using street view data.

Table S1 Summary statistics of individual-level variables (stratified by the presence of kidney failure)

| Variable | Mean (SD)/ Numbers (%) | |  |
| --- | --- | --- | --- |
|  | With kidney failure | Without kidney failure | p-value |
| Gender |  |  | 0.002^a^ |
| Male | 27(71.05) | 975(46.08) |  |
| Female | 11(28.95) | 1141(53.92) |  |
| Age (years) | 58.94(0.89) | 59.40(6.58) | 0.433^b^ |
| Educational attainment |  |  | 0.000^a^ |
| Primary school and below | 8(21.05) | 784(37.05) |  |
| High school and above | 30(78.95) | 1332(62.95) |  |
| Annual household income |  |  | 0.003^a^ |
| < 30,000 Yuan | 10(26.32) | 347(16.40) |  |
| ≥ 30,000 Yuan | 28(73.68) | 1769(83.60) |  |
| Career |  |  | 0.000^a^ |
| White-collar worker | 27(71.05) | 414(19.57) |  |
| Others | 11(28.95) | 1702(80.43) |  |
| Controlled low calorie and low fat diet |  |  | 0.083^a^ |
| Yes | 5(13.16) | 132(6.24) |  |
| No | 33(86.84) | 1984(93.76) |  |
| Physical activity behaviour |  |  | 0.286^a^ |
| Active | 11(28.95) | 791(37.38) |  |
| Inactive | 27(71.05) | 1325(62.62) |  |
| BMI (kg/m2) | 25.01(0.91) | 25.37(3.69) | 0.598^b^ |

^a^ Student's t-tests

^b^ Chi-square tests


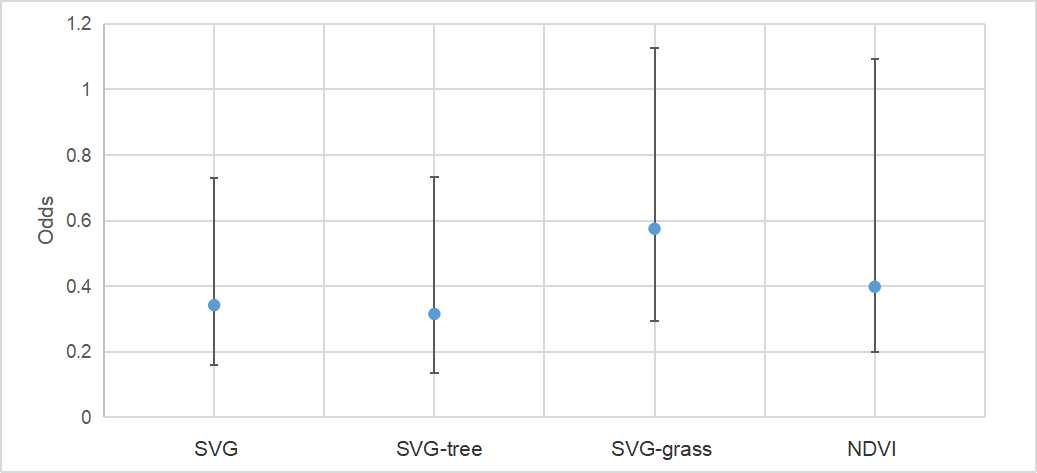

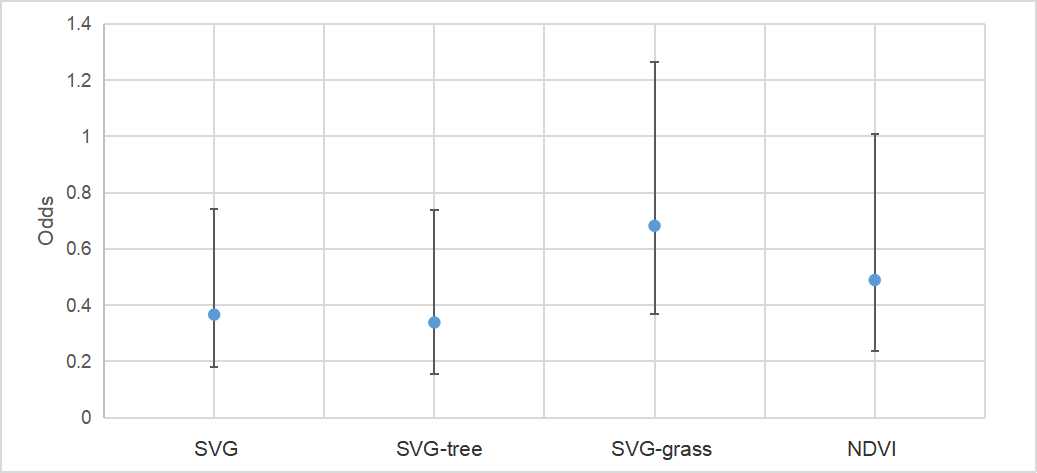


1. (b)

Fig S1 Results of the multilevel models used to examine the association between green space exposure and kidney failure (sensitivity analysis).


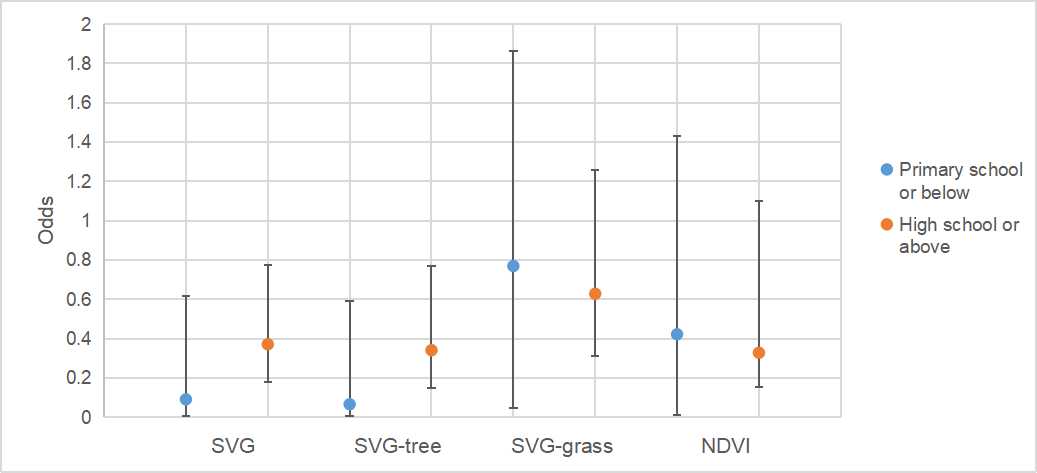


Fig S2 Results of the multilevel models used to examine the association between green space exposure and kidney failure (stratified by educational attainment)


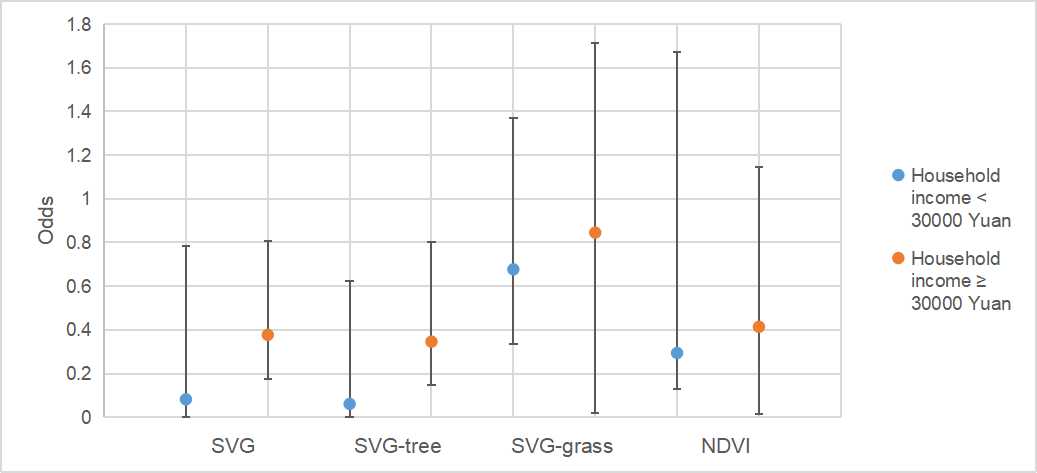


Fig S3 Results of the multilevel models used to examine the association between green space exposure and kidney failure (stratified by household income)
